# An evolving story of the metastatic voyage of ovarian cancer cells: cellular and molecular orchestration of the adipose-rich metastatic microenvironment

**DOI:** 10.1038/s41388-018-0637-x

**Published:** 2018-12-19

**Authors:** Takeshi Motohara, Kenta Masuda, Matteo Morotti, Yiyan Zheng, Salma El-Sahhar, Kay Yi Chong, Nina Wietek, Abdulkhaliq Alsaadi, Eli M Carrami, Zhiyuan Hu, Mara Artibani, Laura Santana Gonzalez, Hidetaka Katabuchi, Hideyuki Saya, Ahmed Ashour Ahmed

**Affiliations:** 1 Ovarian Cancer Cell Laboratory, Weatherall Institute of Molecular Medicine, University of Oxford, Headington, Oxford OX3 9DS UK; 2 Nuffield Department of Women’s and Reproductive Health, Women’s Centre, John Radcliffe Hospital, University of Oxford, Headington, Oxford OX3 9DU UK; 3 Faculty of Life Sciences, Department of Obstetrics and Gynecology, Kumamoto University, 1-1-1 Honjo, Chuo-ku, Kumamoto City, Kumamoto, 860-8556 Japan; 4 Division of Gene Regulation, Institute for Advanced Medical Research, Keio University School of Medicine, 35 Shinano-machi, Shinjuku-ku, Tokyo, 160-8582 Japan

**Keywords:** Ovarian cancer, Cancer microenvironment

## Abstract

Metastasis is a complex multistep process that involves critical interactions between cancer cells and a variety of stromal components in the tumor microenvironment, which profoundly influence the different aspects of the metastatic cascade and organ tropism of disseminating cancer cells. Ovarian cancer is the most lethal gynecological malignancy and is characterized by peritoneal disseminated metastasis. Evidence has demonstrated that ovarian cancer possesses specific metastatic tropism for the adipose-rich omentum, which has a pivotal role in the creation of the metastatic tumor microenvironment in the intraperitoneal cavity. Considering the distinct biology of ovarian cancer metastasis, the elucidation of the cellular and molecular mechanisms underlying the reciprocal interplay between ovarian cancer cells and surrounding stromal cell types in the adipose-rich metastatic microenvironment will provide further insights into the development of novel therapeutic approaches for patients with advanced ovarian cancer. Herein, we review the biological mechanisms that regulate the highly orchestrated crosstalk between ovarian cancer cells and various cancer-associated stromal cells in the metastatic tumor microenvironment with regard to the omentum by illustrating how different stromal cells concertedly contribute to the development of ovarian cancer metastasis and metastatic tropism for the omentum.

## Introduction

Epithelial ovarian cancer is the leading cause of death among malignancies of the female genital tract, and the past few decades have witnessed only slight improvements in survival outcomes for patients with ovarian cancer [1–3]. The high mortality rate of ovarian cancer is attributed, in part, to its nonspecific symptoms, which usually appear when the cancer has progressed to an advanced stage, and the lack of effective screening strategies to detect it at an early stage [4, 5]. Despite ongoing efforts to organize and unify screening programs for ovarian cancer [6, 7], only a limited number of women are diagnosed before the cancer spreads beyond the ovaries (stage I). In the initial stage, a large number of patients can be cured by conventional therapeutic strategies. However, despite advances in surgical techniques and intensive combination chemotherapy approaches, the survival rate substantially decreases after ovarian cancer has metastasized to pelvic organs, such as the uterus, fallopian tube, bladder, and rectum (stage II); metastasized across the pelvic cavity to abdominal organs, such as the omentum, small intestine, and retroperitoneal lymph nodes (stage III); or metastasized beyond the peritoneal cavity to distant parenchymal organs, such as the liver, and lung (stage IV) [1, 5, 8]. Although many patients with advanced-stage disease initially respond to a combination of taxane and platinum-based chemotherapy, some chemoresistant cancer cells can persist in metastatic sites and remain dormant, eventually causing relapse [2, 3, 9].

The majority of ovarian cancers are epithelial in origin and are histopathologically classified into the following five main types: high-grade serous carcinoma, low-grade serous carcinoma, endometrioid carcinoma, clear cell carcinoma, and mucinous carcinoma [10]. These cancer types are inherently diverse diseases that are characterized by differences in precursor lesions, molecular mechanisms of carcinogenesis, patterns of progression and metastasis, responses to chemotherapy, and clinical outcomes [11–14]. In the early twenty-first century, a series of morphological and molecular genetic studies led researchers to propose a dualistic model of ovarian carcinogenesis that divided ovarian cancer into two groups: type I and type II [15, 16]. High-grade serous carcinoma, which is a prototypical type II tumor, is the most common and extremely aggressive subtype and contributes primarily to the poor prognosis of ovarian cancer patients [5, 17, 18]. Because of the high metastatic potential of high-grade serous carcinoma, a large proportion of patients are diagnosed at an advanced stage with multiple intraperitoneal disseminated tumors. Furthermore, a marked predilection for the adipose-rich omentum as the site of metastasis can be observed [4, 5]. Considering that most ovarian cancer-related deaths are directly attributable to the development of metastatic disease, an in-depth understanding of the cellular and molecular aspects of ovarian cancer metastasis is crucial to overcome this life-threatening disease [19–21].

Over a century ago, the English surgeon Stephen Paget proposed the “seed and soil” hypothesis, which stated that the pattern of metastasis is not random and that the development of cancer metastasis depends on the crosstalk between particular cancer cells “the seeds” and a specific organ microenvironment “the soil” [22, 23]. Since then, extensive efforts have been made to evaluate the reciprocal interactions between cancer cells and tumor microenvironments, which are heterogeneously composed of different cell types, including fibroblasts, endothelial cells, adipocytes, various bone marrow-derived cells, such as myeloid-derived suppressor cells, mesenchymal stem cells (MSCs), and macrophages [24, 25]. Researchers have shown that a host of stromal cells within the tumor microenvironment possess the ability to not only promote the progression of the primary tumor but also influence all aspects of the metastatic process by dynamically communicating with cancer cells via direct interactions and paracrine signaling networks [24, 26]. In fact, ovarian cancer cells acquire the capacity to recruit a variety of stromal cells via the secretion of numerous soluble factors to establish a specialized tumor microenvironment. Various activated cancer-associated stromal cells then coevolve with cancer cells and govern the multistep metastatic cascade of ovarian cancer in this milieu [27]. Given that the omentum is the preferred site of ovarian cancer metastasis and represents a central player in creating a metastatic tumor microenvironment in the intraperitoneal cavity [28, 29], knowledge of the cell-biological and molecular mechanisms that regulate the elaborate interactions between ovarian cancer cells and stromal components within the orchestrated tumor microenvironment will shed light on novel therapeutic strategies for the improvement of survival outcomes in patients with advanced ovarian cancer.

Here we present an overview of the mechanisms underlying ovarian cancer metastasis and metastatic predilection for the adipose-rich omentum in the intraperitoneal milieu. We also highlight the recent advances in the understanding of the reciprocal interactions between ovarian cancer cells and various cancer-associated stromal cells in the omental metastatic microenvironment, with a special focus on the functional roles of adipocytes, MSCs, fibroblasts, and macrophages in association with a complex array of tumor-promoting signaling molecules.

## Ovarian cancer metastasis and organ tropism for the adipose-rich omentum

Ovarian cancer is characterized by rapid proliferative growth, peritoneal disseminated metastasis, and malignant ascites within the intraperitoneal cavity [20, 30]. The biological mechanisms that regulate ovarian cancer metastasis are distinctive and notably different from the classic mechanisms of hematogenous metastasis that frequently occurs in most solid cancers [31]. In certain cancers, such as breast, lung, liver, colorectal, and prostate cancers, the metastatic cascade represents a multifaceted process that includes local invasion, intravasation, survival in circulation, extravasation, and metastatic colonization at distant metastatic sites. Thereby, cancer cells frequently encounter with a number of environmentally grueling challenges [32]. In ovarian cancer, peritoneal disseminated metastasis seems to be easier because of the lack of anatomical barriers around the primary ovarian cancer in the milieu of the peritoneal cavity [33, 34]. Upon successful detachment from primary tumor, ovarian cancer cells can survive by forming multicellular spheroids with some stromal components, which float in the specific microenvironment of ascitic fluid, and then metastasize predominantly to the omentum and peritoneum via a direct mechanism. This phenomenon leads to multiple disseminated tumors within the intraperitoneal cavity [20].

In the metastatic process, the phenomenon wherein cells of particular types of cancer preferentially colonize only a limited subset of target organs is defined as “organ tropism,” which is classically referred to as the “seed and soil” hypothesis [22, 25]. Previous studies and clinical observations have highlighted the fact that ovarian cancer cells inherently possess a distinct metastatic tropism for the adipose-rich omentum and peritoneal surfaces [20, 35]. The omentum is a large fold of visceral peritoneum covering the intestine anteriorly in the abdominal cavity [29]. As a central modulator of peritoneal homeostasis, the omentum regulates inflammation, controls fluid exchange, promotes angiogenesis, acts as a source of stem cells and various immune cells, and stores and supplies lipids in the peritoneal milieu [29, 36]. The metastatic behavior of ovarian cancer with tropism for the omentum indicates that the intraperitoneal metastatic microenvironment centering on the omentum plays a critical role in driving metastatic progression of ovarian cancer cells.

Emerging evidence about the hematogenous metastasis of ovarian cancer cells with a strong predilection for the omentum has prompted a rethink of the theory of ovarian cancer metastasis [37]. Ovarian cancer is generally assumed to metastasize preferentially via direct transcoelomic dissemination instead of the hematogenous route [20, 34]. However, a recent paper by Pradeep et al. demonstrated a novel mechanism of hematogenous metastasis to the omentum by using a parabiosis mouse model that involves the surgical union of two mice to allow the sharing of blood circulation [37]. The authors revealed that host mice-derived circulating ovarian cancer cells were able to first metastasize to the omentum of the conjoined guest mice and subsequently spread to the peritoneum and other abdominal organs across the intraperitoneal cavity. Mechanistically, elevated levels of ErbB3, which is a member of the epidermal growth factor (EGF) receptor (EGFR) family, in circulating ovarian cancer cells and neuregulin 1, which is an ErbB3 ligand, in the omentum have been shown to play functional roles in the hematogenous spread of ovarian cancer with metastatic tropism for the omentum [37]. With regard to the metastatic routes of ovarian cancer cells to the omentum, further studies are required to explore the clinical relevance of these observations and clarify the precise mechanisms involved in ovarian cancer peritoneal metastasis.

## Orchestration of the metastatic tumor microenvironment in the adipose-rich omentum

The tumor microenvironment represents a dynamic milieu that involves a complex network of interactions between cancer cells and various stromal components and plays a crucial role in cancer cell survival, proliferation, invasion, and metastasis [24, 38]. A number of studies of late years have provided a theoretical foundation to clarify the active crosstalk between ovarian cancer cells and several types of stromal cells in the omental metastatic tumor microenvironment [27, 28, 39]. The omentum harbors a variety of stromal cell types, including adipocytes, MSCs, fibroblasts, and macrophages, under steady-state physiological conditions. In a malignant setting, these stromal cells can be dynamically converted to distinct cancer-associated stromal cells via cancer-derived mediators, which attract cancer cells to the omentum and support their rapid metastatic growth [27, 40]. A recent study by Pearce et al. demonstrated the complexity and dynamic nature of matrisome remodeling in the omental tumor microenvironment during development of ovarian cancer metastasis by measuring gene expression, matrisome proteomics, cytokine and chemokine expression, cellularity, extracellular matrix organization, and biomechanical properties. These multi-layered gene and protein profiles of the metastatic site provided insight into the comprehensive mechanisms underlying the establishment and deconstruction of the metastatic tumor microenvironment of the omentum [41]. Therefore, uncovering the cellular and molecular organization of the intraperitoneal metastatic microenvironment with a central focus on the omentum will yield novel insights into the evolving biology of ovarian cancer metastasis and provide a useful framework for the further investigation and development of molecular therapies targeting the tumor microenvironment in patients with ovarian cancer.

### Adipocytes and metabolic tumor microenvironment

**Fig. 1:**
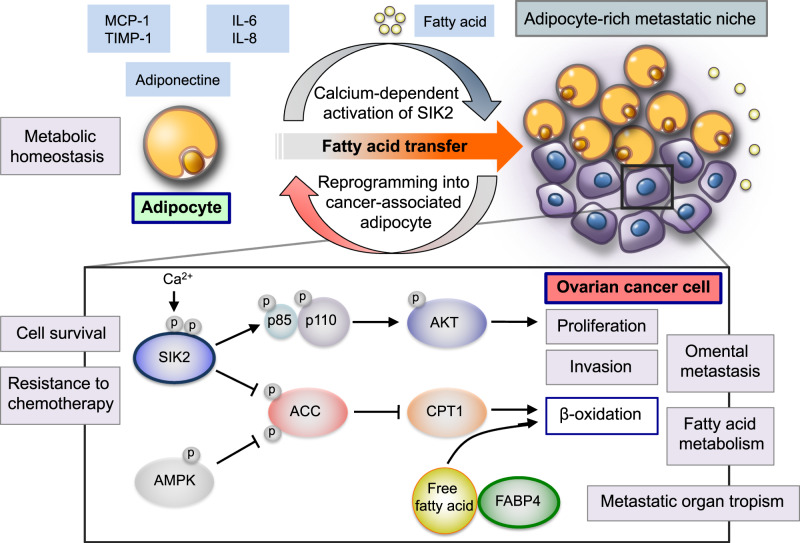
Adipocytes represent a central player in the creation of the metabolic tumor microenvironment in the omentum during ovarian cancer metastasis. Adipocytes are reprogrammed into cancer-associated adipocytes by cancer-derived mediators. These activated adipocytes release a wealth of lipids and various adipokines, including IL-6, IL-8, MCP-1, and TIMP-1, which contribute to the establishment of the omental metastatic niche for ovarian cancer. FABP4, which is an intracellular chaperone for free fatty acids, regulates lipolysis in adipocytes and β-oxidation in ovarian cancer cells, and plays a crucial role in the interaction of ovarian cancer cells with adipocytes, promoting omental metastasis. In addition, omental adipocytes induce the calcium-dependent activation and autophosphorylation of SIK2 in ovarian cancer cells and activate cancer cell proliferation through the PI3K/AKT pathway. SIK2 can increase ovarian cancer cell fatty acid oxidation by augmenting AMPK-induced ACC phosphorylation and activating CPT1 during peritoneal disseminated metastasis. Thus, the highly orchestrated crosstalk between ovarian cancer cells and omental adipocytes induces metabolic synergies by reprogramming fatty acid metabolism and tumor-promoting signaling pathways that enhance the proliferation, invasion, and metastatic progression of ovarian cancer cells with specific metastatic tropism for the omentum

Adipocytes are the most important and abundant cellular component of omental and peritoneal tissue [29] (Fig. 1). As the master regulator of lipid storage and through the production of a panel of adipokines and endocrine molecules, adipocytes contribute to a variety of biological functions, including cellular metabolism, inflammation, and cancer development, under both physiological and pathological circumstances [36, 42]. Emerging evidence has indicated that bidirectional interactions between cancer cells and adipocytes cause the reprogramming of adipocytes into “cancer-associated adipocytes,” [43] and these activated adipocytes are able to release large amounts of lipids, adipokines, tumor-promoting factors, and hormones, which represent a major part of the metabolic tumor microenvironment and promote aggressive cancer growth and metastatic progression [36, 44]. It is interesting to note that Chau et al. recently demonstrated that both omental and peritoneal adipocytes arise from Wilms’ tumor 1 (WT1)-expressing methothelial progenitor cells during their development by using a lineage-tracing model [45]. These findings raise the possibility that omental and peritoneal adipocytes, which share a common embryological background, concertedly contribute to the establishment of the intraperitoneal metastatic microenvironment and ovarian cancer dissemination by inducing metabolic changes that occur in interacting cancer cells and adipocytes.

In recent years, researchers have started to identify the molecular basis for the establishment of the intraperitoneal metastatic tumor microenvironment associated with the interplay between ovarian cancer cells and adipocytes, as well as the metastatic tropism for the omentum [35, 46]. Nieman et al. showed that omental adipocytes are intimately associated with the ovarian cancer metastatic cascade, including invasion, migration, and homing to omental tissue [47]. After undergoing coevolution with ovarian cancer cells, activated omental adipocytes increase the secretion of various types of adipokines, such as interleukin-6 (IL-6), IL-8, monocyte chemoattractant protein-1 (MCP-1), tissue inhibitor of metalloproteinase-1 (TIMP-1), and adiponectine, and these adipokines promote the multistep process of ovarian cancer dissemination with metastatic tropism for the omentum. It should be noted that omental and peritoneal adipocytes can transfer fatty acids to ovarian cancer cells by direct interactions, thus resulting in the alteration of intracellular metabolic activities within cancer cells and the rapid metastatic growth of cancer cells by energy generation via β-oxidation [47].

Fatty acid binding protein 4 (FABP4) is an intracellular chaperone for free fatty acids, which is induced during adipocyte differentiation, and plays an important role in the regulation of lipid metabolism, inflammatory responses, and angiogenesis in several types of solid cancers [48, 49]. In patients with high-grade serous carcinoma, a high-level FABP4 in primary ovarian cancer tissue was found to be significantly associated with increased incidence of residual disease after primary debulking surgery, indicating the possibility that FABP4 expression is an important decision factor for beneficial surgical interventions [50]. Furthermore, the expression of FABP4 was found to be greater in omental metastatic tumors, particularly at the adipocyte-cancer cell interface, than in the primary ovarian tumor. Intriguingly, in a *Fabp4*-knockout mouse model, FABP4 deficiency impaired the metastatic progression of ovarian cancer cells in the peritoneal cavity by altering lipid availability, indicating that FABP4 is a crucial modulator of ovarian cancer metastatic progression in the omentum [47]. Moreover, recent work identified the additional functional roles of FABP4 in fatty acid metabolism, angiogenesis, and peritoneal dissemination in ovarian cancer [48]. siRNA-mediated FABP4 knockdown in endothelial cells resulted in an increase of fatty acid oxidation and reactive oxygen species, and a decrease in blood vessel formation in in vitro assays. More importantly, in a xenograft ovarian cancer mouse model, the therapeutic delivery of siRNA targeting FABP4 in tumor vessels inhibited the tumor angiogenesis and peritoneal metastasis of ovarian cancer [48]. These findings provide a therapeutic rationale for targeting specific molecules in the metastatic tumor microenvironment [42, 44, 46].

Our research group has previously demonstrated the multifaceted roles of salt-inducible kinase 2 (SIK2), which is a member of the AMP-activated protein kinase (AMPK)-related kinase family [51, 52], in the regulation of various biological and molecular functions in ovarian cancer, including cell survival, proliferation, and sensitivity to paclitaxel [53]. More recently, we found that SIK2 plays an important role in the regulation of ovarian cancer cell metabolism in the context of cancer metastasis to the adipocyte-rich metastatic niche [54]. Among patients with high-grade serous ovarian carcinoma, SIK2 was found to be significantly overexpressed in omental metastatic tumors, with the highest SIK2 level observed at the interface between cancer cells and adipocytes, compared with the level in the corresponding primary tumor. Consistent with these clinical observations, we also showed that the forced expression of SIK2 enhances the metastatic ability of ovarian cancer cells in the intraperitoneal cavity, whereas SIK2 depletion prevents the formation of peritoneal disseminated tumors in in vivo mouse models. Using a co-culture system, we demonstrated that adipocytes isolated from omental tissue induce the calcium-dependent activation and autophosphorylation of SIK2 in ovarian cancer cells and stimulate cancer cell proliferation via the PI3K/AKT pathway. Furthermore, our comprehensive in vitro studies have clearly identified the pivotal role of adipocyte-activated SIK2 in the promotion of fatty acid oxidation via the augmentation of the AMPK-induced phosphorylation of acetyl-CoA carboxylase (ACC) and the activation of carnitine palmitoyltransferase 1 (CPT1) transcription during the metastatic progression of ovarian cancer [54]. These findings indicate that SIK2 plays critical roles in the modulation of fatty acid oxidation and metastatic cancer progression by the orchestration of reciprocal interactions between ovarian cancer cells and adipocytes at the omental metastatic niche. With regard to SIK2-targeted therapeutic strategies, Zhou et al. recently reported the promising effects of the small-molecule SIK2 inhibitor in preclinical models of ovarian cancer [55]. These findings and others pave the way towards testing of SIK2 inhibitors in clinical trials [56].

### Mesenchymal stem cells and pro-tumorigenic microenvironment

**Fig. 2:**
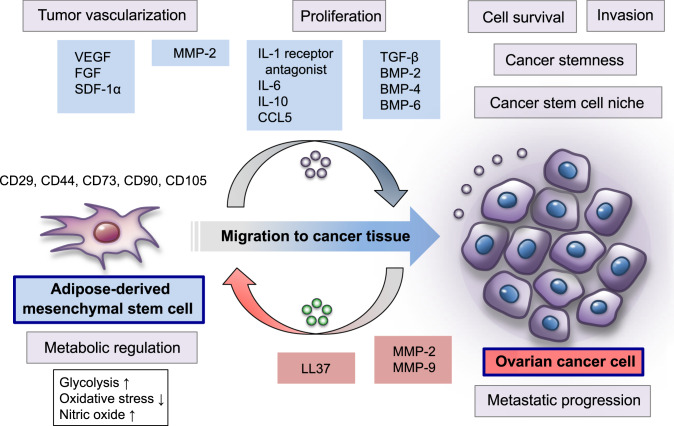
MSCs are an important cellular component of the omental metastatic microenvironment of ovarian cancer. Omental adipose-derived MSCs promote tumor vascularization via the upregulation of VEGF, FGF, and SDF1-α, thereby increasing ovarian cancer cell survival in the metastatic tumor microenvironment. These MSCs can enhance glycolysis and suppress oxidative stress in ovarian cancer cells by regulating nitric oxide levels during the establishment of the omental metastatic niche. In addition, adipose-derived MSCs promote ovarian cancer cell proliferation and metastasis by elevating the expression of MMP2 and MMP-9. Ovarian cancer cells possess the ability to recruit cancer-associated MSCs to the metastatic tumor microenvironment by producing the pro-tumorigenic peptide LL-37. LL37 activates MSCs to secrete a variety of inflammatory and pro-angiogenic factors, including IL-1 receptor antagonist, IL-6, IL-10, CCL5, VEGF, and MMP-2, which are involved in metastatic progression of ovarian cancer cells. Ovarian cancer-associated MSCs, determined by CD44, CD73, and CD90 expressions, play a key role in the formation of the cancer stem cell niche microenvironment in the intraperitoneal cavity, and promote ovarian cancer growth, correlated with enhanced cancer stem cell properties. Mechanistically, these cancer-associated MSCs are involved in the upregulation of the TGF-β superfamily/ BMP family members, especially BMP2, BMP4, and BMP6, and thereby accelerate the aggressiveness of ovarian cancer cells

MSCs are multipotent stem cells with the ability to self-renew and differentiate into cells of mesodermal lineage, such as adipocytes, osteocytes, and chondrocytes, and ectodermal and endodermal lineages [57] (Fig. 2). Although MSCs had been originally identified in the bone marrow, they were later isolated from various different tissues, including adipose tissue, umbilical cord blood, and amniotic fluid [58]. Phenotypically, MSCs represent a heterogeneous cell population and express a specific set of cell surface markers, including CD29, CD44, CD73, CD90, and CD105, but lack essential hematopoietic markers, such as CD14, CD31, CD34, CD45, and CD133 [59, 60]. For over a decade, MSCs have been shown to migrate to the site of wound healing in response to a variety of paracrine and endocrine signals and modify the specific microenvironment, thus accelerating tissue repair and regeneration [57]. Growing evidence has indicated that MSCs are recruited to cancer tissues, which are considered to behave like “wounds that do not heal,” and alter the tumor microenvironment as a crucial mediator, enhancing the aggressive malignant behavior of various solid cancers [57, 61, 62].

A number of studies have been performed to clarify the mutual relationship between ovarian cancer cells and MSCs in the establishment of the supportive metastatic tumor microenvironment [63, 64]. With regard to ovarian cancer metastatic dissemination in the intraperitoneal milieu, one of the most important sources of MSCs is omental adipose tissue that generates adipose-derived MSCs [65, 66]. Functional tumor vascularization is enhanced by omental adipose-derived MSCs via the upregulation of vascular endothelial growth factor (VEGF), fibroblast growth factor (FGF), and stromal cell-derived factor 1-α (SDF1-α), thus resulting in increased cancer cell survival during the formation of the metastatic microenvironment [65]. Omental adipose-derived MSCs can also enhance glycolysis and reduce oxidative stress in ovarian cancer cells by selectively increasing nitric oxide levels by paracrine metabolite secretion, indicating that the metabolic regulation between cancer cells and omental adipose-derived MSCs plays an essential role in the creation of an omental metastatic niche for ovarian cancer [67].

Omental adipose-derived MSCs can alter the defined proteomic profile of ovarian cancer cells in the metastatic tumor microenvironment via paracrine mechanisms, and this capability has been implicated in promoting and sustaining malignant phenotypes, including carcinogenesis, proliferation, migration, apoptosis, and chemoresistance [66, 68]. In an in vitro co-culture model, omental adipose-derived MSCs were found to significantly stimulate the proliferation and invasion of ovarian cancer cells by elevating matrix metalloproteinases (MMPs), which is a family of zinc-dependent endopeptidases, particularly MMP-2 and MMP-9, whereas the inhibition of MMP-2 and MMP-9 was found to partially reduce the tumor-promoting effects of these MSCs. Furthermore, in an in vivo ovarian cancer xenograft model, omental adipose-derived MSCs were found to promote the formation of peritoneal metastasis via the upregulation of MMP-2 and MMP-9 expressions [69].

In terms of the chemotactic signals mediating MSC migration to ovarian cancer tissues, Coffelt et al. revealed the functional role of pro-tumorigenic peptide LL37, which is the C-terminal peptide of human cationic antimicrobial protein 18, in the recruitment of tumor-infiltrating MSCs to the metastatic tumor microenvironment [70]. By using an in vivo migration assay, the authors showed that ovarian cancer-derived LL-37 functions as a chemoattractant for the migration of MSCs into multiple metastatic tumors in the peritoneal cavity; by contrast, the neutralization of LL-37 significantly inhibits the recruitment of MSCs in ovarian cancer tissues, thus resulting in the suppression of metastatic tumor growth. Mechanistically, LL-37 stimulates MSCs to secrete larger amounts of inflammatory and pro-angiogenic factors, such as IL-1 receptor antagonist, IL-6, IL-10, CCL5, VEGF, and MMP-2, and this phenomenon is closely associated with the promotion of ovarian cancer progression and metastasis [70].

On another front, Mclean et al. identified the presence of cancer-associated MSCs, determined by CD44, CD73, and CD90 expressions, in ovarian cancer tissues and malignant ascites and explored the mutual relationship between MSCs and ovarian cancer stem cells [71]. By using an in vivo mouse model, the authors revealed that cancer-associated MSCs enhance ovarian cancer growth that is associated with increasing cancer stemness, suggesting that ovarian cancer-associated MSCs have the capability to form a specialized cancer stem cell niche microenvironment in the intraperitoneal cavity [72]. Furthermore, the gene expression profiling of cancer-associated MSCs demonstrated the potential roles of transforming growth factor-β (TGF-β) superfamily/bone morphogenic protein (BMP) family members, specifically BMP2, BMP4, and BMP6, in the promotion of ovarian cancer progression. Notably, BMP2 inhibition effectively suppressed MSC-mediated tumor growth by abrogating cancer stem cell properties in a xenograft mouse model, indicating that ovarian cancer-associated MSCs promote an aggressive ovarian cancer phenotype by regulating cancer stem cell function and that BMP2 inhibition may be an attractive therapeutic approach for ovarian cancer metastasis [71].

### Cancer-associated fibroblasts and pre-metastatic niche microenvironment

**Fig. 3:**
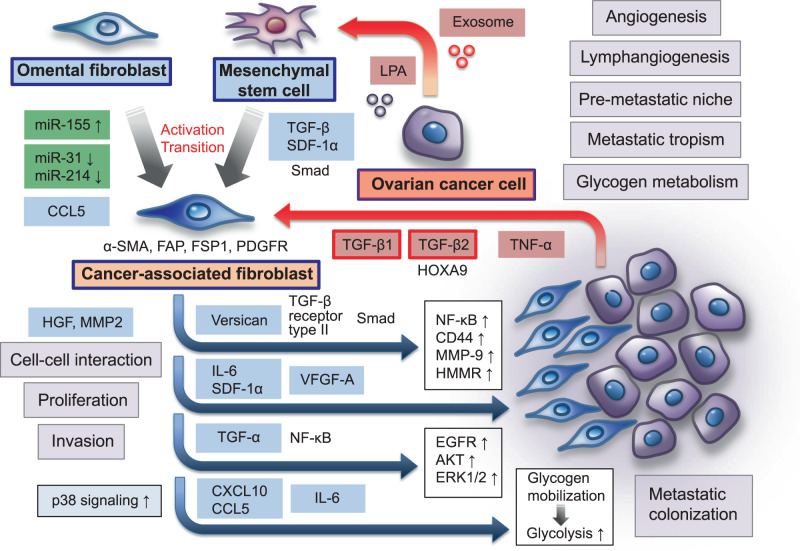
Fibroblasts play a crucial role in the establishment of the omental tumor microenvironment of ovarian cancer. Omental fibroblasts contribute to the creation of a pre-metastatic niche and influence tropism for the omentum and the metastatic colonization of ovarian cancer cells. Ovarian cancer-derived LPA and exosomes stimulate the differentiation of adipose-derived MSCs to CAFs, which are characterized by α-SMA, FAP, FSP1, and PDGFR expressions, by activating TGF-β-related signaling pathways. In addition, ovarian cancer cells can reprogram normal omental fibroblasts to CAFs via the upregulation of miR-155 and the downregulation of miR-31 and miR-214. This promotes tumor growth through increased secretion of CCL5. Ovarian cancer-derived TGF-β1, TGF-β2, and TNF-α, are involved in stimulating the production of various tumor-promoting factors, such as versican, IL-6, SDF-1α, and VEGF-A, in the metastatic tumor microenvironment. In particular, CAF-derived TGF-α promotes the metastatic colonization of ovarian cancer cells via the activation of EGFR, AKT, and ERK1/2 signaling pathways. Metastasizing ovarian cancer cells can activate p38 signaling in omental CAFs, and CAF-derived p38-regulated cytokines and chemokines, including IL-6, CXCL10, and CCL5, induce cancer cells to metabolize glycogen through glycolysis, which mediates energy production and accelerates the aggressiveness of ovarian cancer cells. Thus, in the intraperitoneal metastatic microenvironment, CAFs coevolve with ovarian cancer cells and govern the metastatic cascade, including the adhesion, proliferation, invasion, and colonization of metastatic sites

Fibroblasts display distinct cellular phenotypes depending on their surrounding microenvironment, and activated fibroblasts in cancer tissues have been termed as “cancer-associated fibroblasts” (CAFs) [73] (Fig. 3). Over the years, extensive biological studies have demonstrated that CAFs are the major cellular components of the tumor microenvironment in both primary and metastatic tumors and contribute to the regulation of a series of crucial steps in malignant progression, including cancer initiation, proliferation, invasion, and metastasis, by producing various types of cytokines, chemokines, growth factors, and matrix-degrading enzymes [38, 74]. CAFs can be distinguished from their normal counterparts by altered expressions of markers, such as α-smooth muscle actin (α-SMA), fibroblast activation protein (FAP), fibroblast-specific protein 1 (FSP1), and platelet-derived growth factor receptor (PDGFR) [73]. However, the cellular origin of CAFs and mechanisms underlying the reprogramming of normal fibroblasts into CAFs have remained largely unresolved.

With regard to the heterogeneous origin of CAFs during the metastatic spread of ovarian cancer cells, it has been recognized that CAFs are derived from several different sources according to their supportive microenvironment [75]. Jeon et al. revealed that lysophosphatidic acid (LPA), which is a small bioactive phospholipid, in the malignant ascites of advanced ovarian cancer patients stimulates the differentiation of adipose-derived MSCs to CAFs via the activation of the TGF-β/Smad signaling pathway in the intraperitoneal tumor microenvironment [76]. Similarly, ovarian cancer cell-derived exosomes, which are a class of secreted bilipid membrane vesicles, are able to cause adipose derived-MSCs to acquire the phenotypic and functional characteristics of CAFs by elevating the expressions of TGF-β and SDF-1, which can promote the invasive phenotype of ovarian cancer cells [77]. On the other hand, Mitra et al. demonstrated that ovarian cancer cells have the ability to reprogram normal omental fibroblasts to CAFs via the action of microRNAs (miRNAs), such as miR-13, miR-155, and miR-214; this action can also lead to the upregulation of various chemokines, particularly CCL5, in the omental metastatic microenvironment [78].

In patients with ovarian cancer, clinicopathological evidence indicated that an increase in the number of CAFs identified by α-SMA and FAP expressions is positively correlated with advanced tumor stage, lymph node metastasis, and omental metastasis, which are associated with enhanced angiogenesis and lymphangiogenesis in cancer tissues [79]. Furthermore, despite the lack of detectable metastatic ovarian cancer cells in the omentum, CAFs preliminarily exist in omental tissue in ovarian cancer patients. These findings suggest that before the metastatic colonization of ovarian cancer cells to the omentum, omental fibroblasts contribute to the creation of the specialized microenvironment as a pre-metastatic niche and influence tropism of ovarian cancer cells for the omentum [79, 80].

Over a period of time, a number of in vitro and in vivo studies have revealed the bidirectional interactions between ovarian cancer cells and CAFs associated with the TGF-β-related signaling pathway, and these interactions drive ovarian cancer progression and metastasis [75, 81]. By using a 3D culture model involving the key components of the omental microenvironment, such as fibroblasts, mesothelial cells, and extracellular matrices [82, 83], Cai et al. showed that ovarian cancer cells induce the activation of omental fibroblasts and promote their proliferation by TGF-β1 signaling; in turn, omental CAFs enhance cancer cell adhesion and invasion via the secretion of hepatocyte growth factor (HGF) and MMP-2 [84]. Importantly, in an in vivo ovarian cancer xenograft model, CAFs were found to be remarkably enhance the metastatic ability of ovarian cancer cells with increased metastatic nodules in the peritoneal cavity; however, the inhibition of TGF-β1 signaling attenuated the metastatic dissemination of ovarian cancer. Yeung et al. revealed that ovarian cancer cell-derived TGF-β stimulates the expression of versican, which is a hyaluronate-binding chondroitin sulfate proteoglycan, in CAFs via TGF-β receptor type II and Smad signaling [81]. Upregulated CAF-derived versican can subsequently enhance cancer cell motility and invasion by activating the nuclear factor-κB (NF-κB) signaling pathway and inducing the expressions of CD44, MMP-9, and hyaluronan-mediated motility receptor (HMMR) in the surrounding tumor microenvironment [81]. Moreover, Ko et al. demonstrated the relationship between TGF-β2 and *HOXA9*, which is a Müllerian-patterning gene, in the metastatic progression of ovarian cancer cells in the context of the functional contribution of CAFs [85]. In a mouse xenograft model, the overexpression of *HOXA9* in ovarian cancer cells was found to promote tumor growth and peritoneal dissemination, particularly with regard to omental metastatic tumors, by inducing normal omental fibroblast and adipose- and bone marrow-derived MSCs to acquire the features of CAFs. *HOXA9* are able to induce the production of TGF-β2 in ovarian cancer cells and cancer-derived TGF-β2 acts in a paracrine manner on omental fibroblasts and MSCs to induce the expression of IL-6, SDF-1α, and VEGF-A; this phenomenon is associated with the acquisition of the aggressive phenotype of ovarian cancer cells [85]. Taken together, several lines of evidence indicate that the TGF-β-related signaling pathway plays a crucial role in the crosstalk between ovarian cancer cells and CAFs in the intraperitoneal metastatic tumor microenvironment, suggesting that targeting TGF-β-related signaling may lead to the development of a novel therapeutic strategy against ovarian cancer metastasis [75, 81].

More recently, Lau et al. revealed the role of the tumor necrosis factor-α (TNF-α) –TGF-α–EGFR interaction loop between ovarian cancer cells and CAFs in the development of metastasis to the omental metastatic microenvironment [86]. By using a 3D organoid co-culture model, the authors found that the TNF-α secreted by cancer cells induces the upregulation of TGF-α in CAFs via the NF-κB signaling pathway; thereafter, CAF-derived TGF-α enhances the colony forming ability of metastatic cancer cells through the activation of EGFR, AKT, and ERK1/2 signaling. Intriguingly, in an in vivo ovarian cancer xenograft model, CAFs were found to efficiently promote the metastatic colonization of cancer cells in the intraperitoneal microenvironment, whereas the EGFR tyrosine-kinase inhibitor gefitinib inhibited the metastatic spread of ovarian cancer. These results indicate that EGFR signaling has therapeutic potential for advanced ovarian cancers with disseminated tumors in the peritoneal cavity [86]. Curtis et al. demonstrated that CAFs can promote omental metastasis by inducing changes in glycogen metabolism in ovarian cancer cells in the intraperitoneal tumor microenvironment [87]. They showed that the production of TGF-β1 by ovarian cancer cells activates p38 signaling in CAFs. In turn, CAF-derived p38-regulated cytokines and chemokines, such as IL-6, CXCL10, and CCL5, mobilize glycogen that is associated with fueling glycolysis in cancer cells, increasing proliferation, invasion, and metastasis of ovarian cancer. Furthermore, in in vivo metastasis assay to the omentum, inhibition of p38 signaling in CAFs and treatment of glycogen phosphorylase inhibitor in ovarian cancer cells reduced CAF-stimulated ovarian cancer metastasis, indicating that blocking glycogen mobilization in ovarian cancer cells with glycogen phosphorylase might be a novel therapeutic strategy for metastatic ovarian cancer [87].

### Tumor-associated macrophages and immunosuppressive tumor microenvironment

**Fig. 4:**
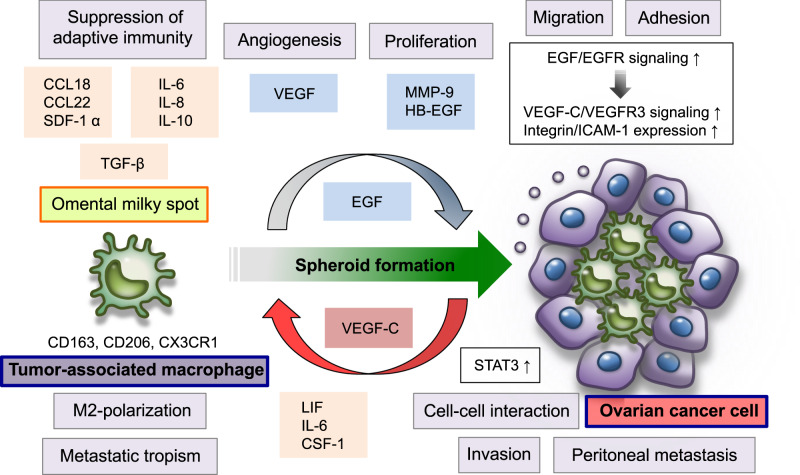
TAMs are involved in the establishment of the inflammatory and immunosuppressive tumor microenvironment during ovarian cancer peritoneal metastasis. Omental milky spots serve as the major sources of intraperitoneal macrophages and play a crucial role in the regulation of metastatic tropism for the omentum. Ovarian cancer cells polarize TAMs to the M2 phenotype, characterized by expressions of CD163, CD206, and CX3CR1, by upregulating LIF, IL-6, and CSF-1 in the specific tumor microenvironment of the intraperitoneal cavity; this microenvironment is modulated by a large number of soluble factors, such as IL-6, IL-8, IL-10, TGF-β, CCL18, CCL22, SDF-1α, VFGF, MMP-9, and HB-EGF. Intraperitoneal M2 macrophages can activate STAT3 signaling pathways in ovarian cancer cells. These pathways are associated with cell–cell interactions between cancer cells and M2 macrophages. Furthermore, these mutual interactions between ovarian cancer cells and macrophages are involved in formation of multicellular spheroids in the malignant ascites of ovarian cancer. EGF secreted by M2 macrophage activates EGFR on ovarian cancer cells, which in turn upregulates VEGF-C/VFGFR3 signaling pathways and enhances integrin/ICAM-1 expression in the intraperitoneal tumor microenvironment, promoting ovarian cancer proliferation, invasion, and intraperitoneal dissemination

Macrophages exhibit a multitude of biological activities in response to microenvironmental stimuli in not only normal physiological conditions but also a variety of disease conditions, including malignancy [88, 89] (Fig. 4). Conventionally, macrophages are classified into M1 and M2 subtypes on the basis of their differentiation status and functional role in the immune system [88]. Classically activated M1 macrophages are stimulated by Th1 cytokine interferon-γ, microbial substrates such as lipopolysaccharide, and toll-like receptor ligands, and they support adaptive immune responses via the production of pro-inflammatory and immunostimulatory cytokines, including IL-1, IL-6, IL-12, IL-23, and TNF-α. Even though there are no specific receptors for identifying M1 macrophages, they commonly express CD68, CD80, and CD86. In contrast, alternative activated M2 macrophages are activated by Th2 cytokines, such as IL4 and IL-13, and they secrete IL−10, TGF-β, and various chemokines, which are involved in tissue remodeling, resolution of inflammation, and cancer progression. Phenotypically, M2 macrophages express specific markers, including CD163, CD204, and CD206 [88, 90].

During cancer development and progression, macrophages are abundantly recruited in tumor tissues, and these macrophages that infiltrate into the tumor microenvironment are referred to as “tumor-associated macrophages” (TAMs) [88, 91]. Generally, TAMs are considered to display the M2-polarized phenotype and are capable of releasing a variety of inflammatory mediators, including cytokines, chemokines, growth factors, and proteolytic enzymes, many of which are crucial components of the immunosuppressive tumor microenvironment that can enhance tumor angiogenesis, invasion, and metastasis [88, 92]. Notably, an increasing amount of evidence has indicated that ovarian cancer cells can polarize TAMs toward the M2 phenotype by upregulating the expressions of leukemia inhibitory factor (LIF), IL-6, and colony stimulating factor-1 (CSF-1), in the intraperitoneal tumor microenvironment [89, 93, 94]. Furthermore, recent clinicopathological studies showed that the expression of the M2-macrophage marker CD163 on intraperitoneal macrophages is correlated with elevated levels of IL-6, and IL-10 in ascitic fluid, which can contribute to poor clinical outcomes in patients with high-grade serous carcinoma [95].

Omental adipose tissue contains clusters of immune cell aggregates called “milky spots,” which are embedded between adipocytes just beneath the mesothelial cell layer [29, 40, 96]. These milky spots primarily include macrophages, B-lymphocytes and T-lymphocytes, and other immune cells, and characteristically act as secondary lymphoid organs [97]. In particular, omental milky spots are considered the major sites for the generation and source of intraperitoneal macrophages that are involved in the regulation of immune responses and inflammation within the intraperitoneal milieu [29, 40, 98]. Importantly, several studies over the past decade have revealed that omental milky spots are the preferred sites of metastatic colonization of cancer cells [40, 99]. A recent study by Clark et al. demonstrated the central role of omental milky spots in the early steps of the metastatic colonization of ovarian cancer [100]. By using a panel of immunodeficient mouse strains in vivo, they found that milky spot macrophages are critical modulators in the creation of the metastatic microenvironment in the omentum that promotes the metastatic colonization of ovarian cancer cells [100].

During the ovarian cancer metastatic process, the intraperitoneal tumor microenvironment, along with the accumulation of malignant ascites, represents a pro-inflammatory and immunosuppressive milieu that includes a large number of spheroid-forming cancer cells, a variety of cell types (e.g., macrophages), and numerous tumor-promoting soluble factors [20, 30, 101]. It is of interest that increased inflammation in the peritoneal cavity can facilitate the peritoneal dissemination of ovarian cancer cells. By using an in vivo xenograft ovarian cancer model, Robinson-Smith et al. demonstrated that the depletion of intraperitoneal macrophages, but not neutrophils or NK cells, significantly reduces the peritoneal metastasis of ovarian cancer. Furthermore, these macrophages partly mediated pro-metastatic effects by enhancing VEGF production in the intraperitoneal milieu [102]. In the context of the complex molecular interplay between ovarian cancer cells and TAMs, the metastatic microenvironment in the intraperitoneal cavity is modulated by a plethora of soluble signaling molecules, including IL-6, IL-8, IL-10, TGF-β, CCL18, CCL22, SDF-1α, VFGF, MMP-9, and heparin-binding EGF (HB-EGF) [90, 92, 103–105]. Furthermore, previous studies have revealed that intraperitoneal M2-polarized macrophages induce the activation of the signal transducer and activator of transcription 3 (STAT3) in ovarian cancer cells and that STAT3 signaling is intimately involved in cell–cell interactions between cancer cells and M2 macrophages, which stimulate the proliferation and metastatic progression of ovarian cancer cells [106].

More Recently, Yin et al. demonstrated that TAMs in the intraperitoneal milieu play a pivotal role in the regulation of multicellular spheroid formation and the transcoelomic peritoneal dissemination of ovarian cancer [107]. They revealed that macrophages are abundantly present in the spheroids floating in the ascitic fluid of patients with advanced ovarian cancer and that an increased number of macrophages in these spheroids are significantly associated with poor prognosis. Interestingly, during the peritoneal dissemination of ovarian cancer, most macrophages are localized in the center of spheroids and gradually gain expression of M2 macrophage markers, including CD163, CD206, and chemokine receptor CX3CR1. These results indicate that spheroid-associated TAMs are mainly polarized to the M2 phenotype and actively contribute to spheroid formation. Furthermore, M2 subtype TAMs secrete a large amount of EGF that activates EGFR on spheroid-associated ovarian cancer cells, which in turn upregulates VEGF-C/VFGF receptor 3 (VEGFR3) signaling and induces integrin/intercellular adhesion molecule 1 (ICAM-1) expression in the surrounding tumor microenvironment as a positive autocrine feedback loop to promote cancer cell proliferation and migration. In an in vivo mouse model, the EGFR inhibitor erlotinib was found to interfere with spheroid formation, proliferation, and metastatic progression of ovarian cancer cells. These findings could be used as a promising therapeutic strategy to inhibit the peritoneal disseminated metastasis of ovarian cancer [107].

## Conclusions and future perspectives

Over the last decade, our comprehensive understanding of the cell-biological and molecular mechanisms that regulate the metastatic cascade and the complex network of interactions between cancer cells and tumor microenvironments has evolved remarkably, and research on cancer metastasis has entered into a stage of considerable progress [108, 109]. In verity, knowledge from a wealth of basic research and clinical studies on cancer metastasis warrants a re-evaluation of current clinical practices and will drive future innovative therapeutic interventions for management of metastatic disease in various types of solid cancers [44, 110].

The biology of ovarian cancer peritoneal metastasis is distinctive because of the extraordinary inflammatory and immunosuppressive milieu of the intraperitoneal cavity, accompanied by the accumulation of malignant ascites [5, 20]. In such specialized circumstances, the adipose-rich omentum plays a principal role in the modulation of the pathological homeostasis of the intraperitoneal milieu and in the creation of the metastatic tumor microenvironment, which are involved in the metastatic progression and organ tropism of ovarian cancer cells [47, 54]. A growing body of evidence has identified the highly orchestrated crosstalk between ovarian cancer cells and their supportive stromal cells in the omental metastatic microenvironment [27, 28]. Notably, the dynamic and reciprocal interplay among these cells are mediated by the secretion of metabolic products including lipids, and by a wide variety of tumor-promoting signaling molecules, such as cytokines, chemokines, and growth factors, leading to omental metastasis of ovarian cancer cells via both transcoelomic and hematogenous routes [37, 46].

Thus, it is essential to advance an integrated understanding regarding the functional roles of cancer-associated stromal cells and their correlated signaling networks in the intraperitoneal metastatic niche centering on the omentum for providing a great opportunity in the management of metastatic ovarian cancer [44]. For the current treatments of ovarian cancer in the metastatic setting, the effectiveness of not only conventional chemotherapy but also molecular-targeted therapy is limited because of the eventual emergence of drug-resistant ovarian cancer cells at metastatic sites. Therefore, further comprehensive investigations are required to identify the roles of the metastatic tumor microenvironment and its constituent stromal cells in the development of drug-resistant in ovarian cancer cells [9, 27, 111]. It is clear that our understanding of the complex chain of the cellular and molecular biology of ovarian cancer metastasis continues to increase steadily. Multiple therapeutic approaches targeting the pathological crosstalk between ovarian cancer cells and the metastatic tumor microenvironment should help in the development of more effective therapeutic approaches for improving the clinical outcomes in patients with ovarian cancer [112]. Our continued voyage to find answers will hopefully open new avenues toward a cure for this life-threatening malignancy.
