# Correction to: An evolving story of the metastatic voyage of ovarian cancer cells: cellular and molecular orchestration of the adipose-rich metastatic microenvironment

**DOI:** 10.1038/s41388-022-02356-0

**Published:** 2022-06-06

**Authors:** Takeshi Motohara, Kenta Masuda, Matteo Morotti, Yiyan Zheng, Salma El-Sahhar, Kay Yi Chong, Nina Wietek, Abdulkhaliq Alsaadi, Eli M. Carrami, Zhiyuan Hu, Mara Artibani, Laura Santana Gonzalez, Hidetaka Katabuchi, Hideyuki Saya, Ahmed Ashour Ahmed

**Affiliations:** 1 Ovarian Cancer Cell Laboratory, Weatherall Institute of Molecular Medicine, University of Oxford, Headington, Oxford, OX3 9DS UK; 2 Nuffield Department of Women’s and Reproductive Health, Women’s Centre, John Radcliffe Hospital, University of Oxford, Headington, Oxford, OX3 9DU UK; 3 Faculty of Life Sciences, Department of Obstetrics and Gynecology, Kumamoto University, 1-1-1 Honjo, Chuo-ku, Kumamoto City, Kumamoto, 860-8556 Japan; 4 Division of Gene Regulation, Institute for Advanced Medical Research, Keio University School of Medicine, 35 Shinano-machi, Shinjuku-ku, Tokyo, 160-8582 Japan

Correction to: *Oncogene* 10.1038/s41388-018-0637-x, published online 19 December 2018

The author name Mohammad Karaminejadranjbar was changed to Eli M. Carrami.

The original article has been corrected.

